# An item response theory evaluation of three depression assessment instruments in a clinical sample

**DOI:** 10.1186/1471-2288-12-84

**Published:** 2012-06-21

**Authors:** Mats Adler, Jerker Hetta, Göran Isacsson, Ulf Brodin

**Affiliations:** 1 Department of Clinical Neuroscience, Karolinska Institutet, Stockholm, Sweden and Psychiatry Southwest Huddinge, Stockholm, SE-14186, Sweden; 2 Department of Learning, Informatics, Management and Ethics (LIME), Karolinska Institutet, Stockholm, Sweden

## Abstract

**Background:**

This study investigates whether an analysis, based on Item Response Theory (IRT), can be used for initial evaluations of depression assessment instruments in a limited patient sample from an affective disorder outpatient clinic, with the aim to finding major advantages and deficiencies of the instruments.

**Methods:**

Three depression assessment instruments, the depression module from the Patient Health Questionnaire (PHQ9), the depression subscale of Affective Self Rating Scale (AS-18-D) and the Montgomery-Åsberg Depression Rating Scale (MADRS) were evaluated in a sample of 61 patients with affective disorder diagnoses, mainly bipolar disorder. A ‘3- step IRT strategy’ was used.

**Results:**

In a first step, the Mokken non-parametric analysis showed that PHQ9 and AS-18-D had strong overall scalabilities of 0.510 [C.I. 0.42, 0.61] and 0,513 [C.I. 0.41, 0.63] respectively, while MADRS had a weak scalability of 0.339 [C.I. 0.25, 0.43]. In a second step, a Rasch model analysis indicated large differences concerning the item discriminating capacity and was therefore considered not suitable for the data. In third step, applying a more flexible two parameter model, all three instruments showed large differences in item information and items had a low capacity to reliably measure respondents at low levels of depression severity.

**Conclusions:**

We conclude that a stepwise IRT-approach, as performed in this study, is a suitable tool for studying assessment instruments at early stages of development. Such an analysis can give useful information, even in small samples, in order to construct more precise measurements or to evaluate existing assessment instruments. The study suggests that the PHQ9 and AS-18-D can be useful for measurement of depression severity in an outpatient clinic for affective disorder, while the MADRS shows weak measurement properties for this type of patients.

## Background

Assessment instruments measuring psychopathology are increasingly being used in the clinical evaluation of depressed patients. Two self-rated assessment instruments used for measurement of depression severity are the depression module from the Patient Health Questionnaire (PHQ9) and the depression subscale of Affective Self Rating Scale (AS-18-D), two relatively new instruments.

The PHQ9 have mainly been evaluated in primary health care and different medical samples showing good psychometric properties in these settings [1-8]. PHQ9 have been suggested as a general measure for depression severity for unipolar as well as bipolar disorder by the task force for the development of the forthcoming fifth edition of the Diagnostic and Statistic Manual (DSM-5) by the American Psychiatric Association (APA) [9]. There is however no studies of PHQ9, to our knowledge, in patients from specialized affective disorder clinics. It therefore seems important to evaluate the properties of PHQ9 in this setting. The AS-18 has been evaluated, using methods from Item Response Theory, in one previous study in a sample of patients with a diagnosis of bipolar disorder, indicating good measurement properties [10].

As comparator we included the well-known observer rated Montgomery-Åsberg Depression Rating Scale (MADRS). Although originally constructed and used for the evaluation of depression in major depressive disorder, MADRS are commonly used for clinical evaluation of depression in bipolar disorder and has become the most widely used severity outcome measure in recent clinical trials of bipolar depression [11,12]. The usefulness of MADRS in measurement of unipolar depression has been investigated in several studies, mostly indicating good measurement properties [13]. The MADRS have also been evaluated in one sample from a clinical trial of bipolar depression, indicating good agreement between clinicians overall assessment of depression severity and changes in MADRS factors [14]. Item response theory (IRT) is a group of modern methods especially developed for the construction and evaluation of assessment instruments, which in most cases are of nominal or ordinal type. IRT-methods have been suggested as a tool for the development of better instruments for the evaluation of depression [15]. Mostly, IRT methods are applied on large studies with the intention to find a representative model and to determine parameters for use when evaluating further individuals (patients, examinees etc.). IRT-methods are also being tested as tools for the evaluation of smaller samples [16]. It would be of great value if IRT-methods could be used for preliminary evaluations of assessment instruments in small samples, for example in the development process of a new instrument (like AS-18) or when an instrument is planned to be used in a new setting (like PHQ9).

The PHQ9 and AS-18-D are used as tools for routine assessment at the Affective disorder outpatient clinic at Karolinska University Hospital Huddinge, where the patient population consists of patients with an affective disorder diagnosis, mainly bipolar disorder type I but also patients with other bipolar diagnoses or major depressive disorder. The instruments are, however, insufficiently evaluated for this type of population.

The aim of this study was to investigate if, in spite of access only to a limited amount of data, successive application of three selected modeling techniques may provide useful information in the evaluation of the two patient-rated assessment instruments PHQ9 and AS-18. Comparisons will be done with the observer rated instrument MADRS.

## Methods

### Study sample

The study was performed at the Psychiatric Clinic Southwest at Karolinska University Hospital Huddinge in Stockholm, Sweden. Patients with a clinical affective disorder diagnosis were opportunistically recruited from the Affective Disorder Outpatient Clinic and inpatient wards when possible during routine clinical work. Sixty-one patients were recruited to the study after having signed a written informed consent form (Regional Ethics committee of Stockholm 04 -752/4). Thirty seven patients were female and 24 male. Age ranged from 17–76 years (average 44, S.D. 13.7). Clinical diagnoses were Bipolar I (N = 37), Bipolar II (N = 8), Bipolar Non Otherwise Specified (N = 8) and Major Depressive Disorder (N = 8). Almost all patients used psychiatric medications.

A recent study of a very large representative sample (n = 13058), using IRT-methods, did reach the conclusion that differences in symptom presentation in bipolar and unipolar depression are subtle (Weinstock et al., 2009). Such subtle differences can not be reliably evaluated with just n = 61. Since we wanted to investigate a clinical sample we decided to keep both unipolar and bipolar patients in the sample.

Recruited patients were given the AS-18 and PHQ9 by the staff and self-ratings were done in a separate room. The self-rating forms were put in a sealed envelope and were blind to the clinicians who did the ratings for MADRS directly after the self-rating procedure. The raters were three experienced clinicians and one resident in psychiatry. The raters had undertaken prestudy training in MADRS-ratings.

### MADRS

MADRS is a ten item interview assessment instrument. It was originally constructed by the selection of the 17 items on the 65 item Comprehensive Psychopathology Rating Scale (CPRS) that were most commonly endorsed by patients in a sample of 106 patients participating in four different trials of antidepressant medication. Ratings on these 17 items were used to select the 10 items which showed the largest changes with treatment and the highest correlation to overall change with medication [11]. Each item in the MADRS has seven category steps with anchoring points at four levels (0, 2, 4, 6). One item focuses on observed sadness. Seven items focuses mainly on reported symptoms of the cognitive and emotional aspects of the depression syndrome (reported sadness, anxiety, concentration difficulties, lassitude, anhedonia, pessimism and suicidality). The two remaining items rate diminished sleep and appetite, two somatic aspects of depression. The time frame for the evaluation of symptoms is not fixed but can be adapted to the purpose of the measurement. In this study the time frame for evaluation was one week prior to the assessment.

### PHQ9

PHQ9 is a nine item patient self rated assessment instrument, based on DSM-IV criteria for depressive episode (anhedonia, depressive feelings, dyssomnia, anergia, appetite problems, negative self-evaluation, concentration problems, suicidality) [2]. It was originally constructed for screening of major depression [1]. It can also be used for measurement of depression severity and for the detection of depression outcome and changes over time [2,4]. In contrast to MADRS, the items for appetite and sleep in the PHQ9 can be scored for both increased and decreased symptom levels. The patients are instructed to rate how much of the time symptoms persisted over the last two weeks. There are four response categories: not at all (0), several days (1), more than half the days (2) or nearly every day (3).

### AS-18-D

AS-18-D is the depression subscale of AS-18, a patient self-rated assessment instrument constructed for assessment of manic, depressive and mixed affective states [17]. In order to differentiate between depression and mania, symptoms that are common to both mania and depression, or difficult to distinguish in a self-report format, were excluded from the assessment instrument (concentration difficulties, decreased sleep and alterations in appetite). Remaining items were intended to capture symptoms specific for depression (increased sleep, hopelessness, motor retardation, depression, anhedonia, anergia, feelings of guilt, slow thinking and suicidality). There are five response categories, graded from zero to four, where respondents can indicate increasing severity of problems caused by the symptoms. The time frame is the week before assessment.

### Statistical methods

The three assessment instruments are constructed with the intention to use the summed score of their constituent items as the outcome measure. This measure is usually assumed to be an appropriate estimate of the severity of a patient’s depression as well as a basis for clinical assessment and for further analysis in scientific studies. The quality and feasibility of such a measure can be evaluated within an IRT-framework. In a small sample perspective, evaluation of an instrument and the use of a sum score as a reasonable measure should preferably be based on a suitable strategy, starting with a robust modelling approach, possibly followed by more structured models to reveal basic characteristics.

In general, assessment instruments in this field are aimed for identifying one underlying latent variable (unidimensionality) by adding items with ordered categories, which requires categories positively correlated to the underlying psychological dimension (monotonicity). The instrument is optimized if the items have the capacity to reliably measure patients at all relevant levels of severity on the dimension (coverage) and the capacity to reasonably estimate their degree of depression. The order of the items, in terms of ‘item severity’ should be similarly conceived by patients at all levels of severity (invariant item ordering, IIO, also named ‘non intersection’). Likewise, there is an inherent intention of equally weighted items (equal item discrimination).

To evaluate the measurement properties of the three assessment instruments we applied an analysis strategy in three steps, using IRT-models of increasing complexity. It should be emphasized that the aim is not to find definite models for further use but rather taking advantage the modelling capabilities to reveal basic structures of the set of items and to some extent point out individual respondents with incoherent answer profiles. As the analysis is carried out on a small sample, a cautious evaluation is needed and special attention should be paid to the sample variation of various estimates.

### A brief layout of the ‘3 step’ strategy

In a first step, the assessment instruments were analyzed using the Mokken non-parametric approach [18]. Such an analysis yields, by use of correlation between ordered categorical variables, a set of *scalability* indexes (H_i_, i = 1,…,I) for items as well as an index, H, for the full assessment instrument. Weak, non-cooperating items can be identified. The total item set scalability, H (which is a weighted mean of the H_i_:s), evaluates the capability of the items to co-operate towards a common over all measure and provides evidence about the degree to which the respondents can be ordered by means of the complete set of items [18]. It will be negatively influenced by deficiencies in *monotonicity*, *unidimensionality* and other sources. There are recommended cut-off levels for the coefficient H indicating whether an assessment instrument has weak, medium or strong measurement capabilities [18]. The Mokken analysis will serve as a first indication of the overall quality of the measurement and to whether further parametric IRT-models will be able to transform the sum scores into a meaningful interval scaled variable. The dimensionality was investigated by the *Automatic Item Selection Procedure* (AISP) in the Mokken R program [19]. Likewise, this program provides explorative analyses as well as graphic options for evaluation of monotonicity and IIO.

The Mokken analysis is the fundamental step in the three step strategy. The estimates of the scalabilities (H and H_i_:s) constitute a basis for decisions about the instrument. Furthermore, the items are subjected to variation that might incidentally influence a decision. An analysis of the sampling variation is of vital importance, especially for items found suspicious by the nonparametric analysis in this first step, in order to not suggest exclusion of potentially contributing items.

We therefore need reliable confidence intervals. Conventional methods for establishing confidence intervals require distributional assumptions which are not available in this case. Therefore boot-strapping methods, based on the actual empirical distribution, are more suitable and have also been shown to work on small samples [20]. The analysis was done in the computer program R [19].

In connection with the Mokken analysis we have also included one of the most well-known estimates of reliability (ie. internal consistency) from Classical Test Theory, the Cronbach’s alpha, which is used as a basis for discussion of possible advantages of the three step strategy compared to classical methods.

Step two: If the Mokken analysis indicated an at least weak scale (H > 0.3 and all H_i_ >0), parametric IRT-models can be used for further analyzing the properties of the instruments. The Rasch Rating Scale Model (RSM) is a parsimonious model, suitable as a starting point for a parametric approach. The RSM considers a common set of category thresholds for all items, meaning that all items have the same set of internal distances between the categories. The RSM also assumes that all items are equally efficient (have the same weight) in discriminating between respondents. A reasonable fit of the RSM would imply that the sum score from an assessment instrument is a sufficient statistic for estimating an interval scaled measure of the respondents’ severity of depression. We consider it important not to omit this step, since the RSM corresponds to the use of the raw sum score. In case of a poor fit to the RSM, however, we will move to a more flexible IRT-model.

Step three: If a model with equal weights is doubtful, the basic RSM can be rewritten and further extended by letting the items have different weights when estimating the model, which now becomes a model within the two-parameter model category (2PL). This means introducing an item specific discrimination parameter that estimates the capacity of the individual item to discriminate between the subjects (within the item’s vicinity) on the scale. The Graded Response Model, GRM, is a suitable form of the 2PL model [21].

The need for item specific weights can be explored by letting the category thresholds catch as much as possible of the available information, i.e. category threshold restrictions are released. The potential set of item specific discriminations is then evaluated by a comparison with an unconstrained model. The ‘ltm’ computer program was used for this purpose [22]. If the test reveals a strong significance, a model with item specific weights, preferably the GRM, will be a reasonable choice.

In general, such a model should be sparsely considered due to the increased number of parameters to estimate and an expected cumbersome statistical uncertainty, due to the limited sample size. However, valuable information might be extracted to elucidate further characteristics of the instrument and how it is perceived by the patients.

In case of a reasonable model fit, person estimates can be calculated on an interval scale together with a measure of precision and thereby also the possibility to reliably differentiate the patients with respect to their severity. Patients with incoherent answer profiles are of particular concern in a small sample study, as they might seriously bias a result. They can be identified by inspection of the relation between the answer profile and the item locations of the applied model and put aside when investigating the instrument.

The match between items and persons on the dimension, the *coverage*, is evaluated by comparing the location of item and person measures on the dimension as estimated by the chosen model. The measurement properties of the instrument are optimized if items are fairly distributed to cover the full range of the respondents’ measures on the dimension. Item Information Functions (IIF) describes how information from the items is distributed over different levels on the dimension. The IIF:s also illustrate the amount of information that can be collected from the individual items. The item information is strongly related to the item weights (discrimination) and an approximate relative magnitude can be calculated.

## Results

Some response categories of the instruments, particularly for MADRS, were sparsely represented (Table 1). For some analyses of the MADRS, categories had to be merged. Applying different ways of joining categories did not seriously change the results.

**Table 1:** Observed frequencies in categories c0,c1,…

	**c0**	**c1**	**c2**	**c3**	**c4**	**Missing**		
**AS-18-D1**	26	11	10	9	5	0		
AS-18-D2	11	12	11	15	11	1		
AS-18-D3	25	9	12	12	3	0		
AS-18-D4	14	8	14	17	8	0		
AS-18-D5	16	7	14	14	9	1		
AS-18-D6	14	10	13	15	9	0		
AS-18-D7	17	8	11	12	13	0		
AS-18-D8	22	11	15	7	5	1		
AS-18-D9	37	12	8	2	1	1		
	**c0**	**c1**	**c2**	**c3**	**Missing**			
PHQ9:1	13	16	13	17	2			
PHQ9:2	18	12	8	23	0			
PHQ9:3	13	12	12	24	0			
PHQ9:4	11	20	10	20	0			
PHQ9:5	25	12	13	11	0			
PHQ9:6	18	10	13	20	0			
PHQ9:7	17	8	12	24	0			
PHQ9:8	26	15	10	10	0			
PHQ9:9	38	16	3	4	0			
	**c0**	**c1**	**c2**	**c3**	**c4**	**c5**	**c6**	**Missing**
MADRS1	24	12	12	2	7	3	1	0
MADRS2	26	8	15	6	2	0	0	4
MADRS3	15	7	15	17	6	1	0	0
MADRS4	26	3	10	7	13	0	2	0
MADRS5	40	4	9	3	5	0	0	0
MADRS6	12	6	12	9	21	0	1	0
MADRS7	24	6	18	3	9	1	0	0
MADRS8	29	6	15	7	3	0	1	0
MADRS9	17	3	19	13	8	0	0	1
MADRS10	38	6	10	3	4	0	0	0

### Step 1. The nonparametric approach

The Mokken scalability analysis showed fairly well behaved instruments for AS-18-D and PHQ9. All item scalabilities were above the recommended cut off of 0.3 and the overall scalability H were above the recommended 0.5 for a strong scale (Table 2) [18]. However, in such a small study, the variability might be quite substantial and has to be investigated. The boot-strapping procedure indicated that the lower bound for a 90% confidence interval (C.I.) for AS-18-D as well as PHQ9 was above the cut-off for a medium performing instrument (medium: 0.4 ≤ H < 0.5) [18]. The weakest item in AS-18-D, item 1 with H_1_ = 0.326, might have been considered acceptable just by chance. By a resampling procedure, the estimated 90% C.I. interval was [0.19, 0.44], which is convincing for the item as contributing with respect to the construction of the underlying dimension. The same can be said about PHQ9:5, for which H_5_ = 0.385, 90% C.I. [0.24, 0.54].

**Table 2:** Scalabilities in the Mokken nonparametric analyses

**Item scalabilities, Hi**						
**Item**	**scalab.**	**Item**	**scalab.**	**Item**	**scalab.**	scalab*.
AS-18-D1	0.326	PHQ9:1	0.584	MADRS1	0.469	0.514
AS-18-D2	0.590	PHQ9:2	0.603	MADRS2	0.442	0.476
AS-18-D3	0.460	PHQ9:3	0.481	MADRS3	0.419	0.449
AS-18-D4	0.576	PHQ9:4	0.478	MADRS4	**0.103**	
AS-18-D5	0.557	PHQ9:5	0.385	MADRS5	0.338	0.359
AS-18-D6	0.491	PHQ9:6	0.588	MADRS6	0.358	0.370
AS-18-D7	0.543	PHQ9:7	0.567	MADRS7	0.281	0.349
AS-18-D8	0.513	PHQ9:8	0.400	MADRS8	0.388	0.456
AS-18-D9	0.582	PHQ9:9	0.468	MADRS9	0.371	0.410
				MADRS10	0.257	0.332
Total H	0.513		0.510		**0.339**	**0.415**
Boot-strapping C.I.	[0.41, 0.63]		[0.42, 0.61]		[0.25, 0.43]	[0.31, 0.51]
Cronbach´s alpha	0.89		0.88		0.81	0.84
no. of obs.	57		59		56	56

* Scalabilities calculated with MADRS4 deleted.

For MADRS however, the scalability analysis showed a scale with three weak items (scalability <0.3). Moreover, MADRS4 showed some negative pair wise scalabilities (H_4,j_ j = 7,8,10, values not shown). The same result emerged when the scale was reduced by merging response categories to x = (0,0,1,1,2,2,2). Also the overall scalability H of 0.339 [0.25, 0.43] was weak, indicating that the items have difficulties to form a main underlying dimension (weak: 0.3 ≤ H < 0.4) [18]. The low scalability of MADRS4 implies that the item might not contribute at all to the measurement. A 90% boot-strapping C.I. resulted in [−0.05, 0.30]. A hypothesis of H_4_ =0 was statistically investigated by repeated permutations of this item’s responses. 500 resampled data sets after permutation yielded a 90% C.I. of [−0.14, 0.19], saying that the observed H_4_ = 0.103 is well within the sample variability for a H_4_ = 0. However, MADRS4 was kept in the model for the subsequent analyses as the aim was to evaluate the instrument as it is constructed.

Analyses of a possible multidimensionality in the three instruments by AISP did not indicate any immediate concern about marked second dimensions.

Before further steps to parametric Rasch models or beyond were taken, item monotonicity and non-intersection was evaluated by the restscore method within the Mokken nonparametric analyses [18]. In general, the minimum size of a restscore group is set to n = 15, which in essence means a low, a median and a high score group.

The AS-18-D showed no violation against the requirements of monotonicity and non-intersection. In the PHQ9 instrument, no obvious violations were recognized against monotonicity but a statistically significant, however small, violation against non-intersection between PHQ9:2 and PHQ9:8 were found. The MADRS showed a few small violations against monotonicity for MADRS6 and a number of significant violations against non-intersection were recognized in five items (item 1, 3, 6, 9 and 10). Although such violations are quite possible to be observed just by chance in such a limited study, this should be a warning for the further analyses. On the whole, these findings did not overthrow the Rasch model as a possible valid tool for revealing the characteristics of the instruments.

### Step 2. Rasch rating scale model (RSM)

Basic RSM:s were then set up for the three instruments according to the intention of the instruments. The RSM, however, indicated large differences concerning the item discriminating capacity and was therefore considered not suitable for the data (data not shown).

### Step 3. A graded response model

Since the analysis in the Rasch model indicated deficiencies in discrimination and model fit, a graded response model was applied, adding parameters for differences in item weights (2PL GRM) [23]. The results from these analyses, based on a model with common category thresholds, are shown in Table 3 and 4. The discrimination parameters are now adequately estimated within the model and the large variation in item discriminations, indicated in the previous step, was confirmed.

**Table 3:** Item locations as estimated by the 2PL GRM

**Item**	**Item location**	**Corrected location to person mean = 0**	**S.E.**
**AS-18-D1**	0.902	0.766	0.354
AS-18-D2	0.096	−0.039	0.164
AS-18-D3	0.677	0.541	0.324
AS-18-D4	0.479	0.344	0.182
AS-18-D5	0.299	0.164	0.221
AS-18-D6	−0.134	−0.270	0.303
AS-18-D7	0.241	0.106	0.187
AS-18-D8	0.635	0.499	0.276
AS-18-D9	1.542	1.406	0.239
PHQ9:1	0.055	−0.013	0.189
PHQ9:2	−0.094	−0.162	0.168
PHQ9:3	−0.266	−0.334	0.244
PHQ9:4	−0.114	−0.182	0.221
PHQ9:5	0.551	0.483	0.284
PHQ9:6	0.067	−0.001	0.174
PHQ9:7	−0.393	−0.461	0.225
PHQ9:8	0.728	0.660	0.273
PHQ9:9	1.417	1.349	0.263
MADRS1	0.160	0.133	0.198
MADRS2	0.588	0.561	0.222
MADRS3	−0.252	−0.279	0.284
**MADRS4**	1.832	1.805	1.372
MADRS5	1.161	1.134	0.361
MADRS6	−0.582	−0.609	0.314
MADRS7	0.641	0.614	0.439
MADRS8	0.052	0.025	0.409
MADRS9	−0.178	−0.205	0.247
MADRS10	1.197	1.170	0.309

**Table 4:** Approximate relative item information and estimated discrimination based on the 2PL GRM model

**Item**	**Rel. info %**	**Discr.**	**S.E(Discr.)**
**AS-18-D1**	**<5**	**0.574**	0.188
AS-18-D2	30	2.845	0.723
AS-18-D3	5	0.923	0.220
AS-18-D4	10	1.256	0.908
AS-18-D5	15	1.751	0.274
AS-18-D6	5	0.861	0.189
AS-18-D7	10	1.313	0.331
AS-18-D8	10	1.102	0.253
AS-18-D9	15	1.710	0.416
PHQ9:1	>25	2.425	0.390
PHQ9:2	10	1.266	0.593
PHQ9:3	5	0.918	0.183
PHQ9:4	10	1.209	0.216
PHQ9:5	5	0.704	0.209
PHQ9:6	15	1.484	0.426
PHQ9:7	10	1.245	0.269
PHQ9:8	5	0.891	0.173
PHQ9:9	10	1.150	0.278
MADRS1	25	1.927	0.719
MADRS2	15	1.425	0.481
MADRS3	15	1.175	0.239
**MADRS4**	**<<5**	**0.273**	0.047
MADRS5	5	0.712	0.209
MADRS6	5	0.659	0.180
MADRS7	5	0.783	0.134
MADRS8	10	1.065	0.155
MADRS9	10	0.898	0.259
MADRS10	5	0.683	0.233

Much of the result from the earlier analyses remains. Weak, less contributing items, which were identified already in the Mokken analyses, were confirmed in the 2PL GRM analyses. AS-18-D1 and MADRS4 are again pointed out as non contributing items. AS-18-D2, AS-18-D5 and AS-18-D9 contribute about 60% of the information in the AS-18-D instrument. PHQ9:1 contributes about 25% in the PHQ9 instrument and MADRS1 + MADRS2 contribute about 40% of the information in the MADRS instrument.

**Figure 1:**
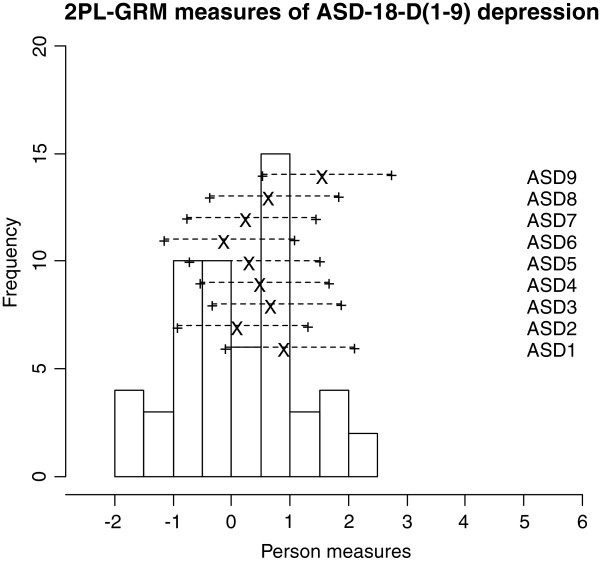
An insufficient coverage of the items compared to the severity of depression in a large part of the sample.

**Figure 2:**
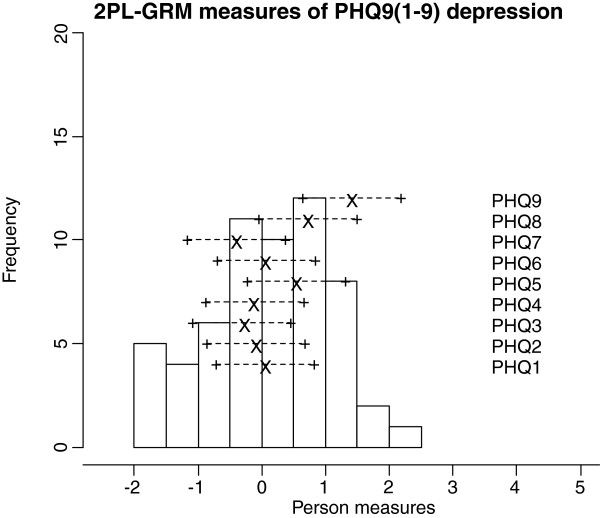
An insufficient coverage of the items compared to the severity of depression in a large part of the sample.

Frequencies of estimated person measures, item locations and category thresholds are shown in Figure 1, 2, 3. These graphs reveal an insufficient coverage of the items compared to the severity of depression in a large part of the sample. The items’ approximate information and their working range are illustrated in Figure 4, 5, 6. The areas under the curves represent the approximate amount of information that can be picked up by the individual items. The curvature tells us that most of the information is obtained from person measures in the middle, around zero. This can be interpreted as a consequence of concentration of the item locations to almost the same range, which also can be seen in Figure 1, 2, 3. This structure is basically the same for all three instruments.

**Figure 3:**
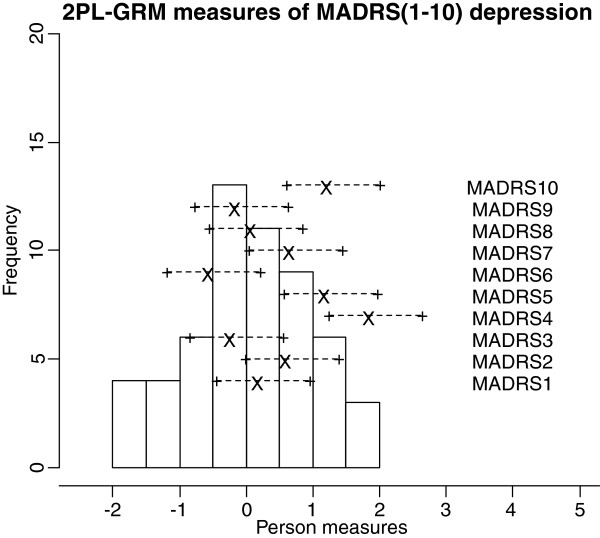
An insufficient coverage of the items compared to the severity of depression in a large part of the sample.

**Figure 4:**
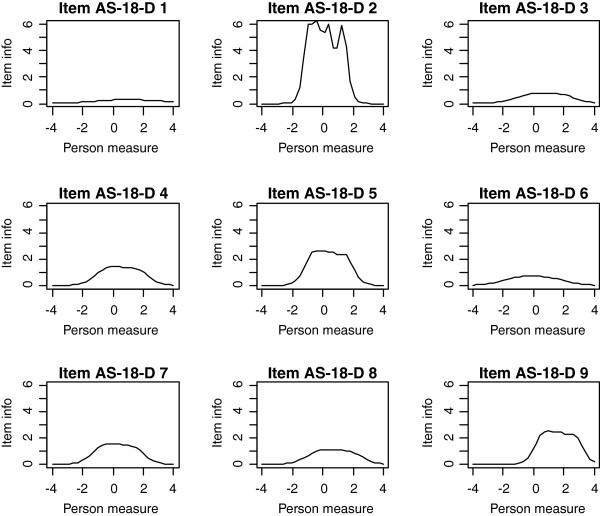
The areas under the curves in Figure 4-6 represent the approximate amount of information that can be picked up by the individual items.

**Figure 5:**
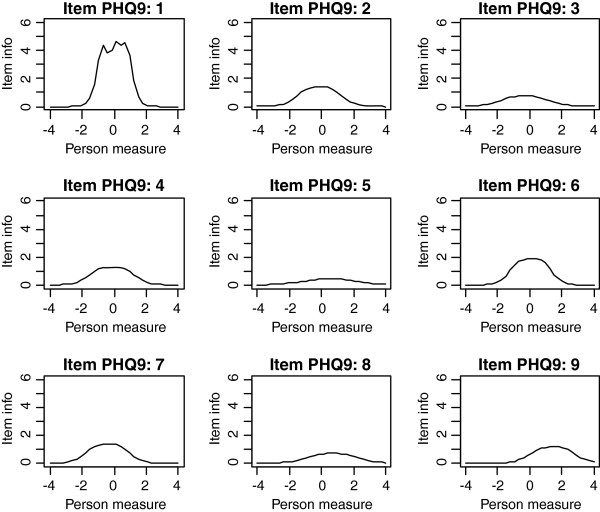
The areas under the curves in Figure 4-6 represent the approximate amount of information that can be picked up by the individual items.

**Figure 6:**
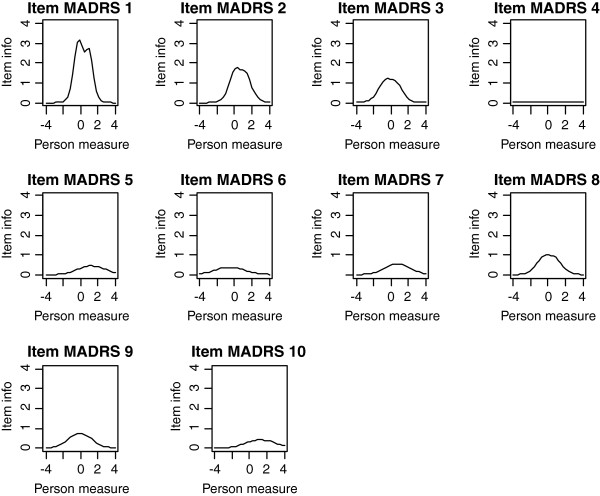
The areas under the curves in Figure 4-6 represent the approximate amount of information that can be picked up by the individual items.

**Figure 7:**
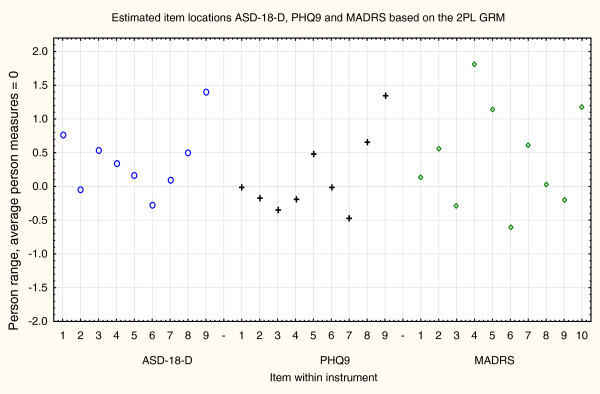
The relationship between the item locations in the three instruments.

The relationship between the item locations in the three instruments is displayed in Figure 7. No item has an optimal measurement level below a depression severity of approximately −0.5.

Extending the models further by item specific category thresholds yielded essentially the same result (not shown). The estimated item weights should be considered as indications, due to the limited sample size.

There were just a few patients showing incoherent answer profiles, in the sense that they looked ‘marked at random’. However, they were not contradictory to the items’ order of severity and were therefore not subject to any special actions.

There were also a few patients, who marked the same category in all items of an instrument. They could be interpreted as ‘apathetic’ or ‘indifferent’. As the interview directed MADRS showed a similar structure for these patients, we considered them as reasonable.

### The dimension as estimated by the three instruments

Clinically, the instruments are considered to reveal the same depression dimension. In the 2PL GRM analysis, each person gets three measures, one from each of the instruments. Even if the sample of individuals is treated as a reference set with the mean set equal to zero in all analyses, we cannot assume that the three measures from AS-18-D, PHQ9 and MADRS are equal. However, if the three variables really measure the same dimension, at least the ranked order of a person would be approximately the same in all three instruments. This means that a person with a high ranking from one of the instruments should be highly ranked on the other two as well. In other words, the correlation between rankings from the three dimensions should be strong.

The results showed that all pairwise correlations were > 0.76. AS-18-D and PHQ9 were more in agreement with each other, about 10% of the persons showed a wide range of intra individual rankings of 15 units or more. ASD-18-D and PHQ9 vs. MADRS showed 17% and 19% respectively. This might look unexpected provided that the three instruments are supposed to measure the same phenomenon. Even if we should not rely too much on the estimated models, such a range can be compared with the message from the models, where the person measures and their SE:s are estimated. We calculated a ranking interval based on a 95% confidence interval for the person measure of one respondent as an illustration of the large possible range of rankings. As can be seen in Table 5, large observed ranking intervals are quite possible and can mainly be interpreted as resulting from the limited precision of the instruments.

**Table 5:** Approximate 95% confidence interval for the rank for person no. 46 based on the GRM model

**Person 46**	**Rank (n=)**	**Rank interval**	**Relative rank %**
AS-18-D	28 (57)	20, 36	35, 63
PHQ9	29 (59)	19, 44	32, 75
MADRS	16 (56)	8, 33	14, 59

## Discussion

Certainly, the main difficulty of this study is the limited sample size. However, the aim was to find a way to reasonably evaluate rating scale type assessment instruments at an early stage (which implies a small sample), and thereby make it possible to introduce improvements or evaluate the appropriateness of their use before a further large scale application. Furthermore, the history tells us that small studies are carried out quite frequently. We therefore suggest this ‘3- step strategy’, which starts out from the use of sum score as the aggregated message from an instrument. It is well known that statistical models are vulnerable when applied on small samples. The ‘sum score model’ is a model based solely on conditions in the assessment instrument – equally weighted items with unit distances between item categories – a fact which is often overlooked. In step 1, the nonparametric approach, basic characteristics are revealed with as few modelling restrictions as possible and form the basis for robust decisions and further analyses. It could be argued that the sample is too small to draw any conclusion regarding the instruments, but the non parametric confidence intervals, based on resampling, for scalability coefficient H tell us that it is unlikely that MADRS is better than a medium performing instrument. Also, the confidence interval together with the permutation test is H for PHQ9 and AS-18-D, however, show that these instruments yield more information than the MADRS regarding the depression severity for this type of patients.

This difference in information quality between the instruments was not found in the analysis using Cronbach´s alpha, where the three instruments showed almost equal estimates. The difference between MADRS with and without the problematic sleep item was not reflected in the alpha. CTT-methods, like the alpha, usually assume continuous interval data with a unique scale parameter and a specified distribution of residuals. Furthermore, most methods assume approximate normal distributions of the residuals from an applied model where the standard deviation is constant over the outcome space. These assumptions are usually not met since data from assessment instruments are of ordinal type [21,24]. Particularly the Cronbach´s alpha have additional problems [25]. First, alpha increases with the number of variables, even if the some of the variables do not contain any information. Second, alpha tends to favor variables near each other on the intended scale, while the aim is to place variables over the whole range (coverage).

The consequences are that Cronbach´s alpha and many other CTT-methods deliver results with unrealistic (falsely high) precision. Unmet optimistic assumptions may lead to sources of error, difficult to grasp. In our opinion IRT-models are better adapted to data of ordinal type and therefore deliver more credible results and realistic estimates of precision.

It has become clear in this study, that the findings in step 1 mostly were verified in the following modelling steps, where further specifications can be found. The Rasch model used in step 2 was shown not to give a reasonable representation of the data. We have, however, not totally excluded the second step in the analysis, since we consider that our three step procedure may be useful in other data sets, even if the present results did not demonstrate strong information of the second step in the analysis of our sample. We consider it unwise to completely exclude step 2. The reason is that a more elaborated model (such as a2PL GRM model) will always show a better fit, but it does not automatically disqualify the more parsimonious Rasch Rating Scale Model. In a small sample situation we have to explicitly declare step 2 as not sufficient, as an extended model with an increased number of parameters might be seen as “overkill”, however necessary to reveal characteristics of the questionnaire.

In step 3, we are well aware that the model cannot be trusted as precise instrument for estimating patient positions, but important information can be collected. Confidence intervals are avoided in this step as they would rely on uncertain model assumptions. Furthermore, model parameter estimates as such are of less importance, since we focus on the instruments.

Using essentially the chain ‘scalability - item location - coverage - item information’ as indicators, we are of the opinion that the conclusions of the study are reasonable in the light of the sample size. However, the study also has other difficulties, not directly connected to the proposed strategy. We used a convenience sample, most patients were on medication and the diagnosis was clinical, which implies a heterogeneous population. Furthermore, the sample consists of patients typical for whom depression assessments instruments should give valid measurement in a clinical setting, which might be seen as an ‘intention to treat’ situation. This limits the possibilities to make firm generalizations about the assessment instruments, but we assert that the suggested method and results in this study are of interest for the evaluation of instruments for clinical use for patients with an affective disorder attending an outpatient clinic.

Taken together, the results indicate that the two self-rating assessment instruments AS-18-D and PHQ9 have the capacity to form a strong unidimensional measure for depression severity for this type of patients. However, the analyses also show that the capacity of the two assessment instruments to reliably estimate the patients’ positions at different levels of severity is limited. The IRT-analysis reveals two main causes of these limitations. First, the severity of depression is measured with high precision only within a narrow range of medium levels. Second, the analysis by GRM demonstrates that many items contribute little to the measurement. If items with low information value were replaced with items with higher information value, covering so far uncovered parts of the depression dimension, the performance of AS-18-D and PHQ9 would improve. Since patients with depression spend so much time in low grade depression it would seem as an important task to improve measurement in the lower range of severity. However, the results do not exclude the PHQ9 in its present form as a useful tool for measurement of depression severity in an outpatient clinic for affective disorder. The AS-18 could be considered an alternative, with roughly equal measurement properties, also having the advantage of including a subscale for manic type symptoms in the same assessment instrument.

It is surprising that MADRS, one of the most frequently used depression instruments for clinical and scientific use in both unipolar and bipolar disorder, show deficiencies in terms of unidimensional scalability. Our findings of low scalability for the MADRS reproduce those of Maier et al. who previously found weak scalability H for the MADRS in unipolar disorder [26]. Allerup, using Rash modelling, found problems with multidimensionality in the MADRS [27], a property which could not be verified in our small sample. The fact that almost all patients in our sample were medicated might have contributed to the finding, since medication previously has been indicated to decrease the dimensionality of the MADRS [28,29].

The explanation for the poor performance of MADRS can be found in three unsuitable items, reduced sleep (item 4), lassitude (item 7) and suicidal thoughts (item 10). Item 4 is especially disturbing and degrades the measurement properties of MADRS. When excluded, the performance of MADRS improves and item 7 and 10 show acceptable scalabilities. A comparison of the approach to sleep disturbances might reveal the cause of the problem. It seems as the MADRS approach, to just include reduced sleep, gives the worst result. The approach of the AS-18-D, to just include increased sleep, performs somewhat better. The PHQ9 approach, to include a broader description of sleeping problems in the item, results in an adequate measurement capacity (“trouble falling or staying asleep, or sleeping too much”). Although the results in this study are from a clinical sample, they can lead to some concern for the usefulness of the MADRS in clinical studies of depression. A weak assessment instrument could lead to failure of a clinical study to detect an existing treatment effect. The practical consequences of improved measurements can be illustrated by calculations showing that antidepressant treatment studies using an improved version of the Hamilton depression Rating Scale (HAM-D-6) would increase the power of a study so it would require approximately one-third less patients than studies using the most commonly used version of the assessment instrument (HAM-D-17) [30,31]. Further studies of the MADRS, using IRT-analysis on both unipolar and bipolar depressed patients, are warranted.

## Conclusions

We conclude that a stepwise IRT-approach, as performed in this study, is a suitable tool for studying assessment instruments at early stages of development. Such an analysis can give useful information, even in small samples, in order to construct more precise measurements or to evaluate existing assessment instruments. The study indicates that the PHQ9 and AS-18-D can be useful for measurement of depression severity in an outpatient clinic for affective disorder, while the MADRS have weak measurement properties for this type of patients. The study also indicates a need for improvement of depression assessment instruments, concerning the ability to reliably differentiate levels of depression, especially mild to moderate. In such an endeavour, IRT-methods for item specific weights and the issue of coverage should be considered.

## Competing interests

MA has received fees for speaking for Eli Lilly, Bristol Meyers Squibb, Sanofi-Aventis and for speaking and preparing manuscripts from AstraZeneca. JH, UB and GI declare no competing interests.

## Authors' contributions

All authors have contributed to the design of the study. MA and GI participated in the data collection. UB was primarily responsible for the statistical analyses. UB and MA drafted the manuscript. All authors read and approved the final manuscript.

## Pre-publication history

The pre-publication history for this paper can be accessed here:

http://www.biomedcentral.com/1471-2288/12/84/prepub
